# A case presenting with a major depressive episode with palilalia and difficulty opening eyes as prodromal symptoms of progressive supranuclear palsy

**DOI:** 10.1002/pcn5.24

**Published:** 2022-06-22

**Authors:** Koji Matsuzawa, Yuichi Yokoyama, Yuichiro Watanabe, Takahiro Wakasugi, Toshiyuki Someya

**Affiliations:** ^1^ Department of Psychiatry Niigata University Graduate School of Medicine and Dental Sciences Niigata Japan; ^2^ Department of Neurology, Brain Research Institute Niigata University Niigata Japan

**Keywords:** antidepressant, botulinum toxin, eyelid opening apraxia, pallido‐nigro‐luysian atrophy (PNLA), progressive supranuclear palsy with predominant postural instability (PSP‐PI)

## Abstract

**Background:**

Progressive supranuclear palsy (PSP) is a neurodegenerative disease and patients with PSP frequently experience depression. However, there have been few reports of patients with major depressive disorder as an antecedent diagnosis of PSP. Here, we report a case who presented with a major depressive episode with palilalia and difficulty in opening his eyes as prodromal symptoms of PSP.

**Case Presentation:**

A Japanese man developed his first major depressive episode at the age of 75 years. At 76 years old, the patient developed palilalia and difficulty in opening his eyes, which worsened with anxiety and agitation. His depression symptoms were not alleviated following treatment with several antidepressants. He gradually became less depressed but more apathetic. Subsequently, he experienced falls and developed nuchal and axial rigidity. Magnetic resonance imaging and ^123^I‐ioflupane single‐photon emission tomography showed predominant midbrain atrophy and postsynaptic striatal dopaminergic degeneration, respectively. He was diagnosed as having symptoms suggestive of PSP at the age of 80 years. The combination of sertraline and aripiprazole reduced his anxiety and agitation. Botulinum toxin treatment provided partial relief for his difficulty in eye opening.

**Conclusion:**

Some patients, such as the current case, develop a major depressive episode at the onset of PSP and present to a psychiatrist. Psychiatrists should therefore be aware of the possibility of a major depressive episode with non‐specific symptoms preceding the onset of the core clinical features of PSP.

## Graphical abstract

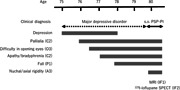

## BACKGROUND

Progressive supranuclear palsy (PSP) is a neurodegenerative disease characterized by ocular motor dysfunction, postural instability, akinesia, and cognitive dysfunction.^1^, ^2^ Few studies have assessed the prevalence of depression in patients with PSP using the *Diagnostic and Statistical Manual of Mental Disorders* (DSM) criteria.^3^, ^4^, ^5^, ^6^, ^7^ It is common for patients with PSP to have broadly defined depressive disorders, including major depressive disorder, dysthymic disorder, adjustment disorder and depressive disorder due to PSP.^3^, ^4^, ^5^, ^6^ However, major depressive disorder is relatively rare.^3^, ^4^, ^6^, ^7^ An autopsy study reported that only 26.2% of patients with definite PSP were accurately diagnosed in the first 2 years of the disease, and that 3% were misdiagnosed with depression.^8^ Yoshida et al.^9^ identified 29 cases with definite PSP in 998 forensic autopsy cases. Of these 29 cases, none were diagnosed with clinical PSP prior to death, but 11 (37.9%) had exhibited signs of depression. These findings suggest that an accurate diagnosis of PSP is difficult in the early stage of the disease and that some patients exhibit depressive symptoms before the onset of the core clinical features of PSP.

There have been few reports of patients with major depressive disorder as an antecedent diagnosis of PSP.^10^, ^11^, ^12^ These case reports had short follow‐up periods and the case presentation was not necessarily detailed. Here, we report a case who presented with a major depressive episode with palilalia and a complaint of difficulty in opening his eyes as prodromal symptoms of PSP. We followed this case for 5 years and we present here the detailed clinical course of the case. We obtained written consent from the patient to publish the features of this case, and the identity of the patient has been protected.

## CASE PRESENTATION

**Figure 1:**
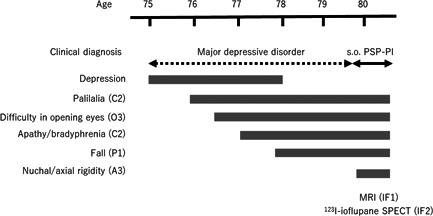
Clinical course of the case. The patient's first major depressive episode occurred at the age of 75 years. At the age of 80 years, the patient was diagnosed as having symptoms suggestive of progressive supranuclear palsy with predominant postural instability (s.o. PSP‐PI) in accordance with the Movement Disorder Society clinical diagnostic criteria for PSP^2^ and the Multiple Allocations eXtinction rules.^13^ Parentheses indicate levels of certainty (1 [highest], 2 [mid], and 3 [lowest]) for the four functional domains of the core clinical features (ocular motor dysfunction [O], postural instability [P], akinesia [A], and cognitive dysfunction [C]) and the supportive features of imaging findings (IF). IF1 indicates predominant midbrain atrophy, as shown in magnetic resonance imaging (MRI). IF2 indicates postsynaptic striatal dopaminergic degeneration, as shown in ^123^I‐ioflupane single‐photon emission tomography (SPECT).

The patient was an 80‐year‐old Japanese man diagnosed as having symptoms suggestive of PSP with predominant postural instability (PSP‐PI). The clinical course of the case is shown in Figure 1. At the age of 75 years, he developed depressed mood, insomnia, psychomotor retardation, feelings of guilt, and suicidal ideation and experienced a loss of interest, energy, and appetite. He was diagnosed with major depressive disorder in accordance with the DSM, 5th Edition criteria at his first psychiatric visit. He was initially treated with 100 mg/day of sertraline, which provided inadequate efficacy, and was subsequently referred to our hospital. At the age of 76 years, his medication was replaced with 30 mg/day of mirtazapine. This and all subsequently prescribed drugs were given with the consent of the patient and his wife. However, he developed anxiety, agitation, and palilalia, in which he repeated words, such as “umm, umm, umm.” One month later, mirtazapine was switched to 60 mg/day of duloxetine. The following month, his suicidal ideation worsened, and the patient was hospitalized for the first time.

His total score on the 17‐item Hamilton Rating Scale for Depression (HAM‐D) on admission was 24. Subsequently, 6 mg/day of aripiprazole was added to duloxetine. Although the patient's palilalia improved, his depressive symptoms persisted. Aripiprazole was increased to 24 mg/day. He developed gait disturbances, which comprised small steps, bradykinesia, and falls. These symptoms resolved when aripiprazole was stopped. His depression was not relieved by 30 mg/day of escitalopram or by 100 mg/day of milnacipran. His total scores on the 17‐item HAM‐D fluctuated between 20 and 22. When 40 mg/day of duloxetine was resumed, his energy and appetite improved; however, his palilalia recurred. Subsequently, he was treated with 100 mg/day of sertraline and 3 mg/day of aripiprazole. His palilalia disappeared, his depressed mood, anorexia, and insomnia improved, and his feelings of guilt and hopelessness decreased. However, he sometimes complained that he was unable to open his eyes, even when his eyes were open. Additionally, apathy and bradyphrenia appeared around the same time. His Mini‐Mental State Examination (MMSE) score was 28. Brain magnetic resonance imaging (MRI) showed mild atrophy of the medial temporal lobe, and ^123^I‐*meta*‐iodobenzylguanidine myocardial scintigraphy revealed that his heart‐to‐mediastinum ratio had not reduced (3.40 and 3.62 for the early and delayed phases, respectively). He was discharged 15 months after admission. His total score on the 17‐item HAM‐D at discharge was 19.

The patient occasionally experienced palilalia and difficulty in opening his eyes. At the age of 77 years, he became unable to operate a personal computer, and at 78 years of age, his gait became slower with smaller steps, and he repeatedly fell. Apathy and difficulty in opening his eyes also worsened, and he was bedridden almost every day. He was re‐hospitalized at the age of 80 years.

**Figure 2:**
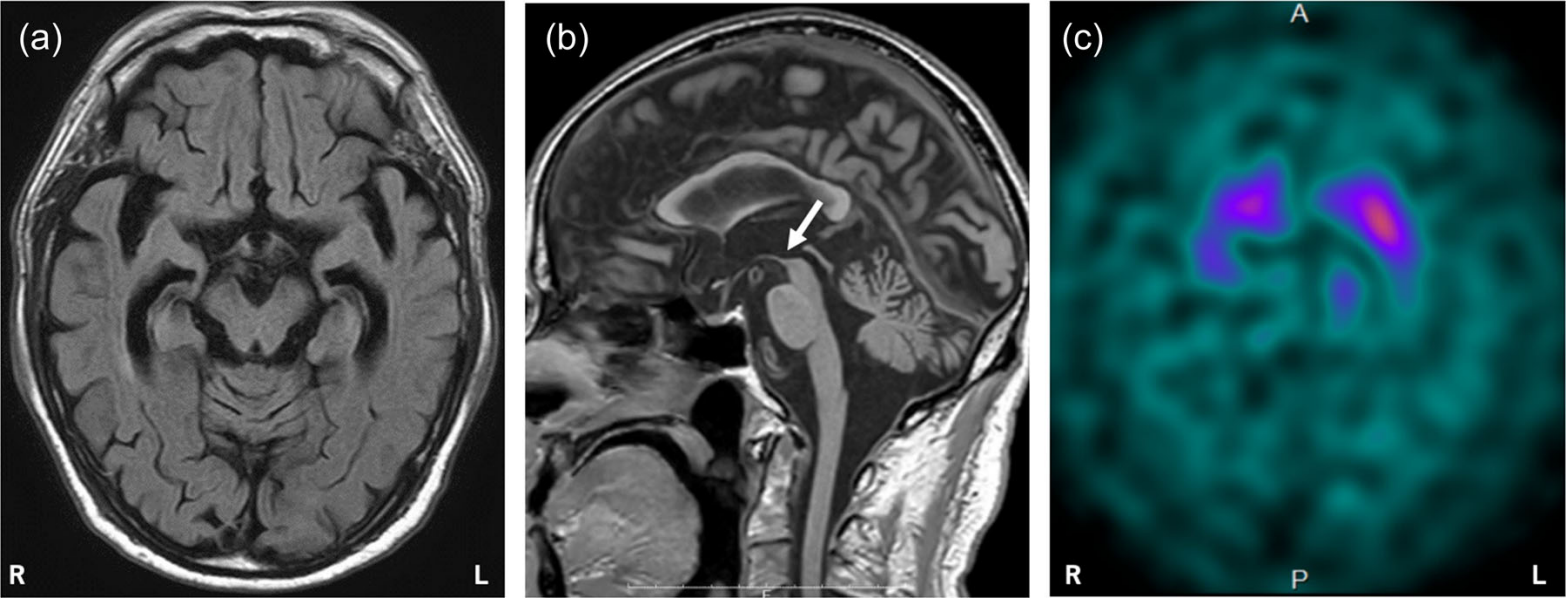
Neuroimaging findings of the patient at the second hospital admission. (a) Axial fluid‐attenuated inversion recovery sequence brain magnetic resonance imaging (MRI) showed mild atrophy of the midbrain and mid‐temporal lobe, including the hippocampus. (b) Sagittal T1‐weighted brain MRI revealed atrophy of the midbrain capsule, which is referred to as the “hummingbird” sign (indicated by the arrow). (c) ^123^I‐ioflupane single‐photon emission tomography shows decreased accumulation in the bilateral striatum.

He had difficulty in opening his eyes (i.e., eyelid opening apraxia). In addition, he repeatedly complained that he was unable to open his eyes, even when his eyes were open. Upward gaze palsy was also noted. These features fulfilled the lowest level of certainty for ocular motor dysfunction (O3).^2^ Repeated unprovoked falls occurred within 3 years after onset of PSP‐related features, which fulfilled the highest level of certainty for postural instability (P1).^2^ We observed gait disturbances, which included small steps and limited arm swing. His neck was slumped and tilted slightly forward, and he exhibited bilateral trunk and limb rigidity. These features fulfilled the lowest level of certainty for akinesia (A3).^2^ His scores for the MMSE and Frontal Assessment Battery were 28 and 13, respectively. He showed palilalia, apathy, and bradyphrenia, which fulfilled the mid‐level of certainty for cognitive dysfunction (C2).^2^ Brain MRI revealed predominant midbrain atrophy (Figure 2a,b), which fulfilled imaging finding 1 (IF1).^2^ Furthermore,^123^I‐ioflupane single‐photon emission tomography showed decreases in the bilateral putaminal specific binding ratios (3.13 and 3.09 for the right and left, respectively; Figure 2c), which fulfilled imaging finding 2 (IF2).^2^ He was diagnosed as having symptoms suggestive of PSP‐PI in accordance with the Movement Disorder Society clinical diagnostic criteria for PSP (MDS‐PSP)^2^ and the Multiple Allocations eXtinction rules.^13^

Although sertraline and aripiprazole were discontinued, gait disturbances and rigidity persisted. Moreover, his palilalia (e.g., “umm, umm, umm”) subsequently worsened with anxiety and agitation. He also repeatedly stated “ouch, ouch, ouch” when he fell. We observed that his difficulty in opening his eyes partially responded to botulinum toxin therapy. However, he repeatedly complained that he was unable to open his eyes. Thus, 100 mg/day of sertraline and 3 mg/day of aripiprazole were resumed, and his palilalia, difficulty in opening his eyes, anxiety, and agitation improved. He was discharged 2 months after his second admission.

## DISCUSSION

The patient's first major depressive episode developed at the age of 75 years (Figure 1). At the age of 76 years, he developed palilalia and difficulty in opening his eyes, which worsened with anxiety and agitation. During this time, we misinterpreted these symptoms as those associated with depression. He gradually became less depressed but more apathetic. Subsequently, we observed other core clinical features of PSP, such as repeated falls and nuchal and axial rigidity. Finally, the patient was diagnosed as having symptoms suggestive of PSP‐PI (O3, P1, A3, C2) at the age of 80 years.

Patients with PSP frequently experience depression^3^, ^4^, ^5^, ^6^, ^14^, ^15^ and are commonly prescribed antidepressants.^14^, ^15^ In this case, depression was not relieved by antidepressants (duloxetine, escitalopram, or milnacipran), with the exception of a combination of sertraline and aripiprazole for reducing anxiety and agitation. According to the consensus statement on best practices in the clinical management of PSP and corticobasal syndrome, selective serotonin reuptake inhibitors may be used; however, tricyclic antidepressants should be avoided.^16^ Nevertheless, in a study of 892 patients with PSP, 231 (26%), 60 (7%), and 114 (13%) patients were prescribed selective serotonin reuptake inhibitors/serotonin‐noradrenaline reuptake inhibitors, tricyclic antidepressants, and other antidepressants, respectively.^14^ There are currently no approved effective treatments for depression in patients with PSP.

Palilalia is a relatively rare speech disorder that has been reported in patients with various neurological disorders.^17^ Palilalia was not noted in 74 patients with multiple system atrophy, whereas it was exhibited by 12 of 39 (31%) patients with PSP.^18^ Kluin et al.^19^ also observed the condition in five of 44 (11%) patients with PSP. Therefore, palilalia is not rare in patients with PSP. As such, it is listed as an example of impulsivity, disinhibition, or perseveration of frontal cognitive/behavioral presentations (C2) in the MDS‐PSP criteria.^2^

In our patient, the complaint of difficulty in opening eyes occurred during the first 2 years of the disease. Botulinum toxin treatment objectively partially relieved this problem, although no subjective improvement was reported. The prevalence of difficulty in opening eyes may be relatively rare during the early stage of the disease and may vary among pathological subtypes of the PSP spectrum. Of 67 patients with clinically diagnosed PSP, one (1%) had lid levator inhibition at the first assessment with a disease duration of 2–4 years, whereas 17 (25%) had lid levator inhibition at the final assessment with a disease duration of 3–9 years.^20^ In our previous study, difficulty in opening eyes was observed in 0 of 22 (0%) patients with PSP, 1 of 9 (11%) patients with pallido‐nigro‐luysian atrophy (PNLA) Type 1, and 0 of 9 (0%) patients with PNLA Type 2 during the first 2 years of the disease.^21^ Moreover, the prevalence of difficulty in opening eyes was significantly higher in patients with PNLA Type 1 than in those with PSP and PNLA Type 2 (5/9 [55.6%] vs. 1/22 [4.5%] and 0/9 [0%], respectively) during the entire disease course. Furthermore, another study reported that eyelid opening apraxia was noted in 2 of 8 (25%) patients with both PSP and PNLA but in 0 of 11 (0%) patients with pure PSP.^22^ Considering these results, we speculate that the pathological diagnosis of our case may be PNLA Type 1 rather than PSP, although an autopsy is required to confirm such a diagnosis.

## CONCLUSION

This case was characterized by a major depressive episode with non‐specific symptoms, including palilalia and difficulty opening eyes as prodromal symptoms suggestive of PSP‐PI. Some patients, such as the current case, develop a major depressive episode at the onset of PSP and present to a psychiatrist.^10^, ^11^, ^12^ Therefore, psychiatrists should be aware of the possibility of a major depressive episode with non‐specific symptoms preceding the onset of the core clinical features of PSP.

## AUTHOR CONTRIBUTIONS

*Writing—original draft, writing—review and editing*: Koji Matsuzawa, Yuichi Yokoyama, and Yuichiro Watanabe. *Writing—review and editing*: Takahiro Wakasugi and Toshiyuki Someya.

## CONFLICT OF INTEREST

All authors declare no conflict of interest.

## DISCLOSURE

Toshiyuki Someya received research support and honoraria from Asahi Kasei Pharma Corp., Astellas Pharma Inc., Daiichi Sankyo Co. Ltd, Dainippon Sumitomo Pharma Co. Ltd, Eisai Co. Ltd, Eli Lilly Japan, K.K., GlaxoSmithKline K.K., Janssen Pharmaceutical K.K., Meiji Seika Pharma Co. Ltd, Mitsubishi Tanabe Pharma Co. Ltd, Mochida Pharmaceutical Co. Ltd, MSD K.K., Otsuka Pharmaceutical Co. Ltd, Pfizer Japan Inc., Shionogi & Co. Ltd, Tsumura & Co., and Yoshitomi Pharmaceutical Industries. openResearch.

## ETHICS APPROVAL STATEMENT

We obtained written consent from the patient to publish the features of the case.

## PATIENT CONSENT STATEMENT

We obtained written consent from the patient to publish the features of the case.

## Data Availability

All relevant data are included in the paper.
